# Determination of Acetone in a Water/Toluene Emulsion in Each Phase Using Raman Spectroscopy with Scattered Light Correction

**DOI:** 10.3390/s26072192

**Published:** 2026-04-01

**Authors:** Erik Spoor, Matthias Rädle, Jens-Uwe Repke

**Affiliations:** 1 CeMOS Research and Transfer Center, Technische Hochschule Mannheim, Paul-Wittsack-Str. 10, 68163 Mannheim, Germany; m.raedle@hs-mannheim.de; 2 Process Dynamics and Operations Group, Technische Universität Berlin, Straße des 17. Juni 135, 10623 Berlin, Germany; j.repke@tu-berlin.de

**Keywords:** disperse phase, continuous phase, optical spectroscopy, emulsion measurement, process control, Raman spectroscopy, process engineering, UV/VIS spectroscopy

## Abstract

**Highlights:**

**What are the main findings?**

- Scattered light correction can be used to measure the concentration of emulsions.
- It is possible to determine the concentration of acetone in both phases whilst they are emulsified.

**What is the implication of the main finding?**

- The scattered light measurement for Raman is an easy solution to correct signal loss due to dispersed droplets, and to determine the concentration of acetone.
- This provides the possibility for simpler process control for the investigation of diffusion processes.

**Abstract:**

Raman spectroscopy is capable of determining the composition of mixtures quantitatively and qualitatively. However, this technology reaches its limits when used to examine liquid dispersed mixtures of substances. In these emulsions, light scattering occurs at the interfaces of the particles and/or droplets, leading to signal losses that make the results impossible to evaluate. Our previous publications have shown, however, that it is possible to quantify the signal losses using a scattered light probe. In an investigation of the water–toluene–acetone emulsion, the acetone concentration could be determined with a root mean squared error of prediction (RMSEP) of up to 1.5 wt%. Based on this method, further analyses are now being carried out to demonstrate that the correction also makes it possible to determine the acetone concentration in each individual liquid phase. First, a ternary diagram is analytically created by establishing stable conditions and separating the phases for individual measurement. In a second step, the samples are measured as a dispersed mixture with the droplets as interfering factors, demonstrating that the same concentration differences can be measured between both phases.

## 1. Introduction

An important area of process analysis technology is the monitoring of chemical reactions using optical measurement technology. Developments in this field are constantly advancing and enable the determination of the concentration of individual components [1,2]. One of these measurement methods is Raman spectroscopy. It can be used to examine mixtures for their qualitative and quantitative properties. A laser with a defined wavelength is focused into a medium, and the Raman effect is induced. This effect causes light to be backscattered which is shifted in its wavelength [3,4]. The shift in wavelength compared to the excitation wavelength (Raman shift) is a molecule group-specific effect and thus allows the identification of the components [5,6]. In addition, in homogeneous fluids, the concentration of the components can be determined from the linear relationship between concentration and measured signal intensity [6,7].

Another advantage of Raman measurement technology is that it can be used non-destructively, noninvasively, and nonintrusively [4,6,8]. The major disadvantage of the method is the use of lasers, which require laser safety measures and pose a risk in terms of explosion protection. Raman spectrometers often operate at powers of up to 500 mW, as this produces high-quality measurement signals that are easy to evaluate. However, some processes require lower signals or systems that have higher sensitivity, thanks to highly sensitive detectors. With the right know-how and equipment, it is therefore possible to measure with power levels well below 10 mW, thereby reducing the risk for health and explosions [9,10,11]. Another limitation of Raman spectroscopy is the presence of fluorescent materials, such as biological samples [12,13]. In such cases, fluorescence interferes with the Raman spectrum, making it difficult or even impossible to interpret.

However, the use of an optical measurement technology becomes more difficult in dispersed systems such as emulsions. Each droplet of an emulsion represents an interface at which a difference in the refractive index occurs. When light crosses the interface, it is refracted, scattered, and reflected, which reduces the power density at the laser’s focal point and causes the backscattered light to be lost on its way to the detector [14,15,16,17,18]. Several solutions to the mentioned problem can be found in the literature and have already been successfully applied. For example, it is possible to perform complex calibrations across several parameters and thus create models that can determine the concentrations. The disadvantage, however, is that a large knowledge of the chemical system must be established and, eventually, the calibration is only valid for the target system in a very specific application [19,20]. Another way to solve the problem is to add additional components that dissolve in only one of the two phases in order to equalize the refractive index and minimize or even completely neutralize the effects of the boundary layers. However, this represents an alteration to the system of compounds and is not suitable for every process [16,21]. Alternatively, phase separation cells can be used. These are installed in a bypass and allow the mixture to separate, so that the phases can then be measured individually. The disadvantage is that additional design effort is required, more complex process control can lead to longer process runtimes, and the measurement does not provide a continuous live signal [22].

A new solution for the problem has already been presented in our previous publications [23,24,25]. A scattered light probe was used for this purpose, which measures the laser light scattered by the Raman probe on the disperse phase, thus enabling the losses to be quantified [23]. The principle is that, in a homogeneous sample, the laser beam passes straight through the sample, and the scattered light probe therefore receives no signal. However, if light scattering occurs at interfaces due to particles or droplets, the scattered light probe detects this. This results in a relationship where an increase in the proportion of the dispersed phase corresponds to an increase in scattered light values. At the same time, the opposite effect occurs in the Raman spectrum, as the Raman signal decreases with an increasing proportion of the dispersed phase. This opposing trend of the Raman signal and scattered light can be correlated to establish a correction function. The measurement principle was also tested with particles of various sizes, and the results showed that both the size and the number of particles are indirectly reflected in the scattered light values. Therefore, no separate knowledge of these parameters is necessary [24]. The basic effects and relationships were demonstrated using glass beads in an ammonium nitrate solution. Based on this study, measurements were carried out on a mixture of water, toluene, and acetone using the same measurement method [25]. Toluene and acetone are both common industrial solvents and are frequently used. The purification process, which involves extraction and rectification, also plays a major role. To better monitor these processes in terms of their concentrations and states of equilibrium, it is important to correct for the interference effects caused by the emulsion. The emulsion is an example application that has already been well documented in the literature in terms of its physical, chemical, and spectroscopic relationships [26,27,28,29]. This system was chosen precisely for this reason, as the results can be verified easily using the extensive data available in the literature. However, the method’s approach is designed to be general enough that it is not limited to the analysis of acetone, toluene, and water, but can also be applied to new applications in future investigations.

The scattered light measurement method ultimately enables the Raman signal to be corrected and the acetone concentration to be determined. Compared to other methods, scattered light correction offers a simple option that can be easily implemented in existing systems and requires only minimal computational and evaluation effort. Furthermore, unlike the refractive index correction mentioned above, no additional substances need to be added to the mixture. Also, no knowledge of the distribution, size, or number of dispersed droplets is necessary, since all this information is indirectly contained in the measured scattered light. However, only the total concentration of the mixture has been investigated so far. The problem becomes more complex when a distinction has to be made between the acetone concentration in the water and toluene phase. Tertiary phase equilibrium diagrams show that there is a difference in concentration in both phases. There is currently no known publication that has determined the concentration of a component differentiated in both phases within a mixed emulsion. Based on the previous approach of scattered light correction, the aim is now to demonstrate that it is also possible to determine the acetone concentrations in these individual phases. This offers an advantage when determining equilibrium states, as diffusion in a fully mixed emulsion can be monitored in real time, thereby providing better insight into the substance system and, consequently, improving process monitoring.

## 2. Materials and Methods

**Figure 1:**
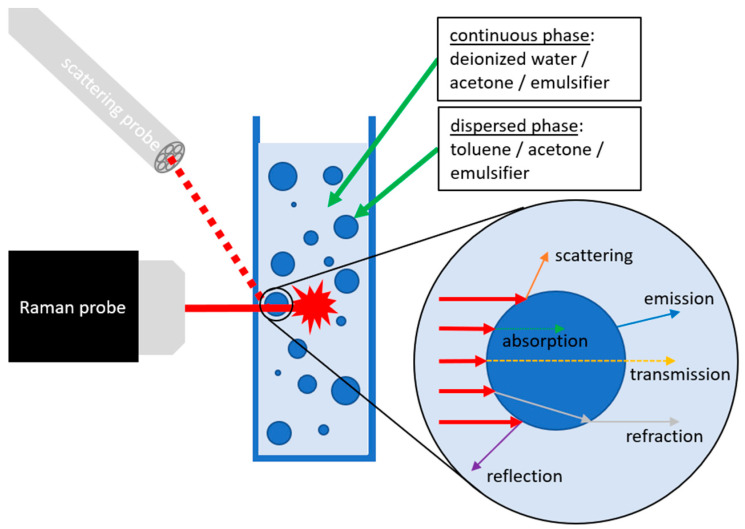
Schematic diagram of the experimental setup with laser and backscatter probe measuring an emulsion of water, acetone, and toluene in a cuvette [25].

The measurement setup shown in Figure 1 consists of a Raman probe, a scattered light probe, and a cuvette with the emulsion to be measured. The Raman probe is connected to a MultiSpec Desk ETH Raman spectrometer from Tec 5 (Steinbach, Germany), and the scattered light probe is connected to an MCS 601 UV-NIR C UV/VIS spectrometer from Zeiss (Oberkochen, Germany). The RPS785/16-5 Raman probe from InPhotonics (Nowrood, MA, USA) is connected to the Raman spectrometer with an LS-LD laser cassette from tec5 and focuses the 785 nm excitation laser with 200 mW into the cuvette. When selecting the laser wavelength, it should be noted that this affects light scattering at the dispersed phase. In general, the influence of the dispersed phase is greater at lower wavelengths. However, an analysis of the emulsion in the previous study showed that the droplet sizes are approximately 1 µm and are thus generally larger than the wavelength used. When using alternative lasers, the relationship to the droplet size must therefore be taken into account in order to be able to compare the data [30,31]. The distance of the probe is set to ensure that the 158 µm-sized focus point, with a focus length of 7.5 mm, is located 2 mm deep in the cuvette from Hellma (Müllheim, Germany). The penetration depth of 2 mm is the theoretical penetration depth in an empty cuvette. During measurement, the penetration depth varies with changes in concentration/refractive index. The minimum refractive index of the samples measured is approximately 1.3 and the maximum refractive index is 1.5. This results in an actual penetration depth ranging from 3.16 to 3.40 mm. As already shown in previous studies, a deeper penetration depth in dispersions would lead to increased signal losses [23]. The resulting Raman signal is recaptured by the same probe and forwarded to the SC-CCD RAMAN detection module (detection range 319–3213 cm^−1^ with increments of 1 cm^−1^ and spectral resolution of 5 cm^−1^) of the Raman spectrometer. The scattered light probe additionally captures the light that is scattered at the interfaces of the droplets. Past measurements have shown that the scattered light increases with increasing portion of the dispersed phase [24,25]. The light from the scattered light probe is transmitted to a Zeiss MCS 601 UV-NIR C spectrometer (detection range 190–1015 nm with increments of 0.5 nm and spectral resolution of 2.4 nm), where it is detected. This spectrometer does not have any specific filters and therefore receives all the light from the sample. However, it is not sensitive enough to detect the Raman signal, which means that only the scattered light from the laser is detected, overlapping all other signals. As a result, the scattered light probe determines how much laser light is scattered at a wavelength of 785 nm.

The emulsion in the cuvette is a mixture of water, toluene, acetone (Carl Roth (Karlsruhe, Germany)), and Polysorbate20 (Merck (Darmstadt, Germany)). Water and toluene are immiscible, and the smaller mass fraction represents the dispersed phase. Acetone is soluble in both phases and represents the target variable to be measured. Polysorbate20 is an emulsifier that is added to all samples at a concentration of 2 wt% to stabilize the emulsion. To obtain the emulsion, the samples were emulsified using a T 25 disperser from IKA-Labortechnik (Staufen im Breisgau, Germany) at 8000 rpm for 10 min. All data points were measured with an integration time of 5 s for the Raman signal and 30 ms for the scattered light signal. Each measurement was recorded 10 times to ensure that the emulsion was stable and the values were reproducible [25].

The measurements were performed at an average room temperature of 24 ± 1 °C in the laboratory. Temperature measurements taken before emulsification and after filling the cuvette show that the temperature increases by approximately 1 to 1.5 °C during handling. The 10 measurements per sample also show no significant signal fluctuations or drifts during laser irradiation. Repeated measurements were also carried out on other days, which resulted in the same Raman intensities, and therefore the overall influence of temperature is considered insignificant.

## 3. Results

**Figure 2:**
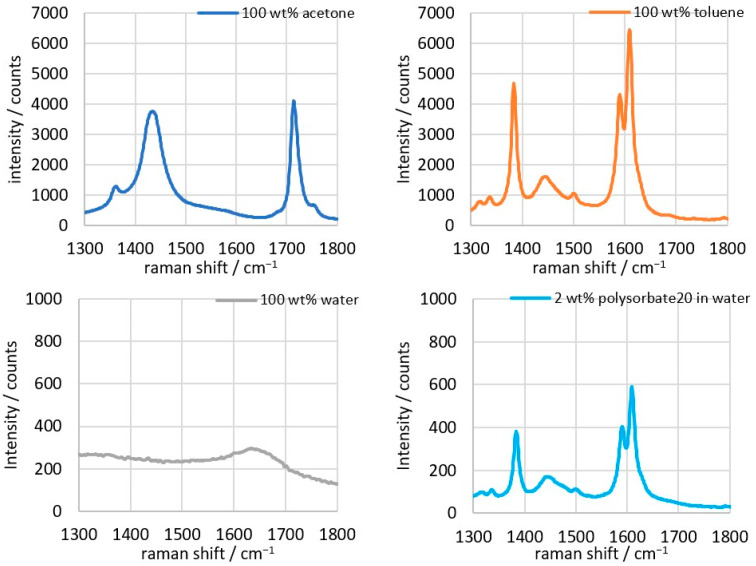
Raman spectra of acetone, toluene, water, and 2 wt% polysorbate in water, in the range from 1300 to 1800 cm^−1^; integration time: 1 s, average of 3 spectra [25].

The characteristic peaks of the individual components have already been analyzed in previous experiments (Figure 2). The range of interest for the measurements performed here is between 1300 and 1800 cm^−1^. The target component whose concentration is to be determined is acetone (C_3_H_6_O), which shows a peak of the C=O group at 1715 cm^−1^ that does not overlap with peaks of the other substances [32]. Toluene (C_7_H_8_) shows its strongest signals through the benzene ring in the range of 790 and 1008 cm^−1^ and thus has no influence on the C=O peak of acetone at 1715 cm^−1^ [26]. Water (H_2_O) has a distinct signal at 3400 cm^−1^ and is therefore well outside the peak range of acetone, meaning it does not cause interference [33,34]. The emulsifier Polysorbate20 (C_58_H_114_O_26_) shows a spectrum comparable to toluene but delivers a measurement signal that is 10 times smaller. This means that all peaks of the emulsifier are overlapped by the remaining components. In addition, the Polysorbate20 content remains constant at 2 wt% in all samples and should therefore not lead to any variations in the signal.

**Figure 3:**
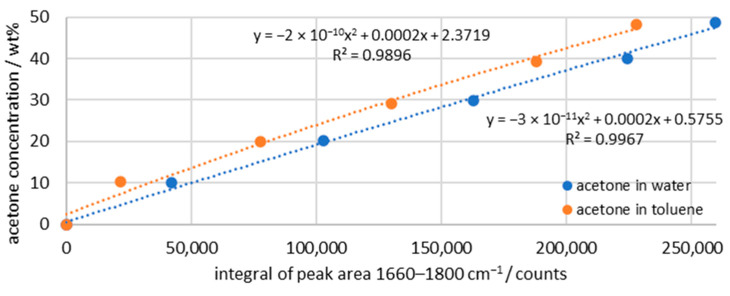
Calibration of peak integrals of the Raman intensity and the acetone concentration; dashed line indicating regression of the data points; integration time: 5 s; average of 10 spectra.

In order to convert the measured intensities of the Raman signal into concentrations later on, trend lines have to be recorded. One trend line is measured with water–acetone and a second trend line with toluene–acetone. For the evaluation, a baseline correction is performed with the two reference points at 1660 and 1800 cm^−1^, and the integral of the same range is calculated. The result is shown in Figure 3 and displays two quadratic functions. The acetone peak in water has a full-width at half-maximum of about 20 cm^−1^, while the peak in toluene is about 6 cm^−1^ smaller. This results in the deviation of the two trend lines shown.

**Figure 4:**
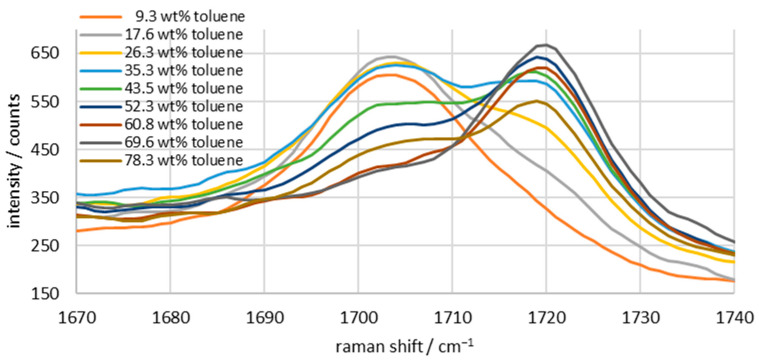
Characteristic acetone peaks at 10 wt% in a water/toluene mixture with varying water/toluene concentrations; integration time: 5 s; average of 10 spectra.

After analyzing the individual components of the mixture, emulsions consisting of all components with varying toluene–water ratios are measured. The acetone content in Figure 4 remains constant at approximately 10 wt%. The toluene content is gradually increased from 9.3 wt% to 78.3 wt%, and the water content is reduced in the opposite direction from 87.8 wt% to 8.8 wt%. The characteristic peak of acetone is in the range from 1670 to 1740 cm^−1^. Figure 2 showed that the peak of pure acetone is a single peak at 1715 cm^−1^. However, Figure 4 shows that the position of the peak depends on the substances in which the acetone is present. Acetone in water forms a peak at approximately 1704 cm^−1^, while acetone in toluene forms a peak at 1720 cm^−1^. Variations in the water–toluene ratio, therefore, result in a double peak whose size and position change depending on the sample. With this knowledge in mind, the signals will be analyzed, and the acetone concentration in the individual phases will be determined in the following. To achieve this, the double peak must first be deconvoluted, then the correction method established in a previous paper [25] must be applied to the peaks, and finally, a second correction must be made based on the phase ratio.

**Figure 5:**
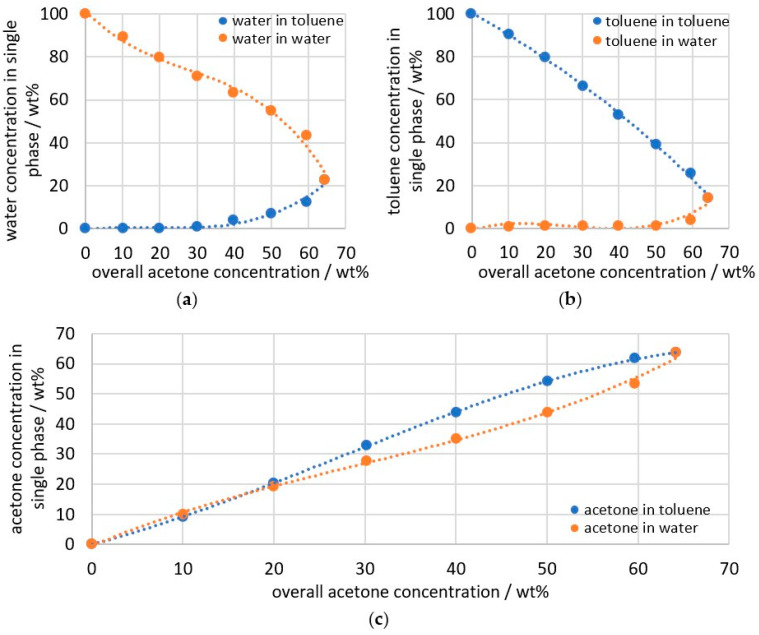
Measurement data from Raman spectroscopy for the water–toluene–acetone emulsion after reaching a stable state and separation into the two individual phases; (**a**) water concentration; (**b**) toluene concentration; (**c**) acetone concentration; dashed line indicating regression of the data points; integration time: 5 s; average of 10 spectra.

Before the double peak is further analyzed, it is necessary to record reference data so that the results can finally be checked for plausibility. Therefore, emulsions are prepared with acetone concentrations ranging from 0 to 65 wt% and a toluene to water ratio of 2:3, without the use of an emulsifier. After the emulsification process, the emulsion separates into a toluene-rich phase and a water-rich phase. The phases can be extracted and measured individually. Figure 5 shows the relationships between the three components in individual phases over the total acetone concentration of the emulsion. Figure 5 shows that toluene, with a solubility of 0.563 g/L [35], is poorly soluble in water. Only at total concentrations of 40 wt% acetone, the water content in the toluene phase and, vice versa, the toluene content in the water phase increase significantly. The two-phase region ends at a composition of 63.4 wt% acetone, 13.9 wt% toluene, and 22.7 wt% water. Figure 5c shows the acetone concentration of the individual phases and is therefore the target value to be determined from the double peak shown in Figure 4. At 10 wt% acetone, the acetone concentration in water is approximately 0.4 wt% above the concentration in toluene. As the acetone concentration increases, this trend reverses, and the acetone concentration in the toluene phase exceeds the concentration in the water phase. The largest difference occurs at 50 wt% acetone, with 54.2 wt% acetone in the toluene phase and 43.8 wt% acetone in the water phase.

**Figure 6:**
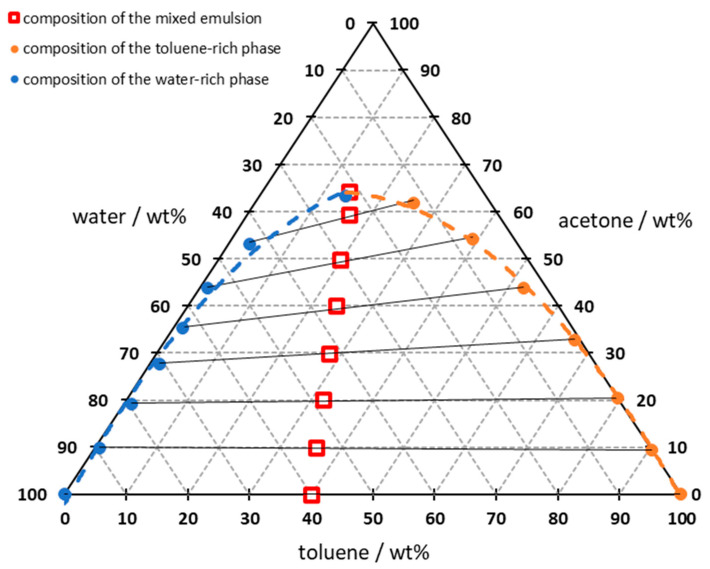
Display of the measurement results, after phase separation, in the ternary diagram with resulting conodes; measured at 24 ± 1 °C; dashed line indicating regression of the data points; solid line connecting data points indicates the conodes; integration time: 5 s; average of 10 spectra.

The results shown in Figure 5 can then be plotted in a ternary phase diagram to illustrate the binodal curve, conodes and miscibility gap (Figure 6). The red squares in the diagram represent the composition of the pre-weighed emulsion. The black solid lines are the conodes of the two-phase region, which point to the measurement points of the water and toluene-rich phases and represent the phase equilibrium. The changing difference in the acetone content of both phases can be visualized by the gradient of the conodes. The resulting diagram shows the same results as diagrams from the literature and is therefore considered to be conclusive [29,36,37].

Sixteen samples were prepared to determine the concentrations of the two-phase region from the mixed emulsion. The samples can be split into four groups, made up of 10, 20, 30, and 40 wt% acetone. With the same acetone concentration, the ratio of toluene to water is 0.00, 0.43, 1.00, and 2.32. The samples were weighed to the third decimal digit, and the readings were used to convert the sample weight to the actual concentration in weight percent. The values in Table 1 are rounded to two decimal places. The weighing error is approximately 0.1% and is considered insignificant for further analysis.

**Figure 7:**
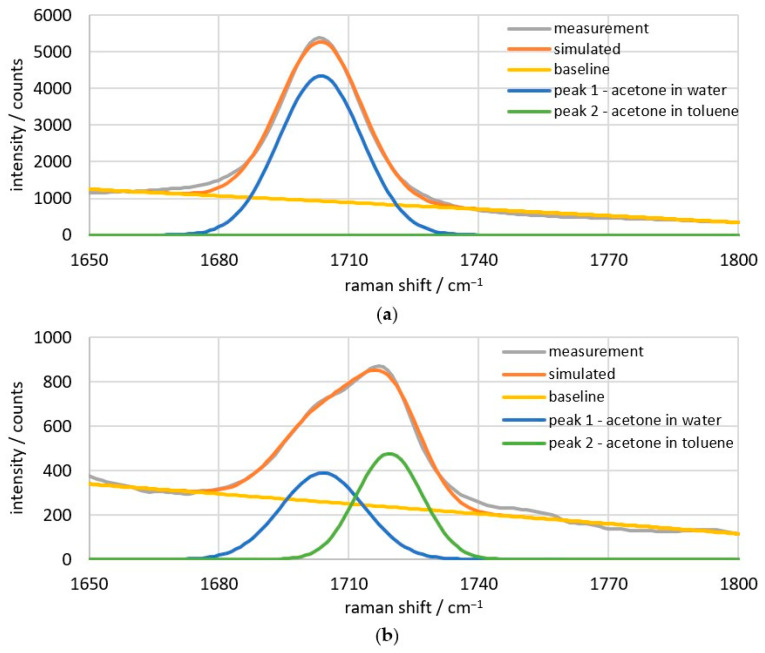
Deconvolution of 20 wt% acetone in (**a**) 78.2 wt% water and (**b**) 38.7 wt% water and 38.9 wt% toluene.

The measured acetone peak is shown for the example of 20 wt% acetone in water (Figure 7a) and a water–toluene ratio of 1:1 (Figure 7b). For all measurement points, a deconvolution is performed according to these examples. For the spectra without toluene content, the deconvolution corresponds in the broadest sense to a simple baseline correction. The baseline is plotted linearly between the reference points at 1660 and 1800 cm^−1^. Figure 7b shows the deconvolution of the double peak into a peak at 1704 and at 1720 cm^−1^, with the same baseline correction. For the peaks, normal distributions are calculated by specifying the position, integral, and spectral width. The position is given, and the other parameters are optimized iteratively. The target value is the smallest deviation of the sum of the baseline and individual peaks (referred to here as “simulated”) from the “measurement” curve. The data in the range from 1680 to 1740 cm^−1^ are primarily relevant for the minimal deviation, as background signals outside this range can otherwise lead to significant deviations from the measurement.

**Figure 8:**
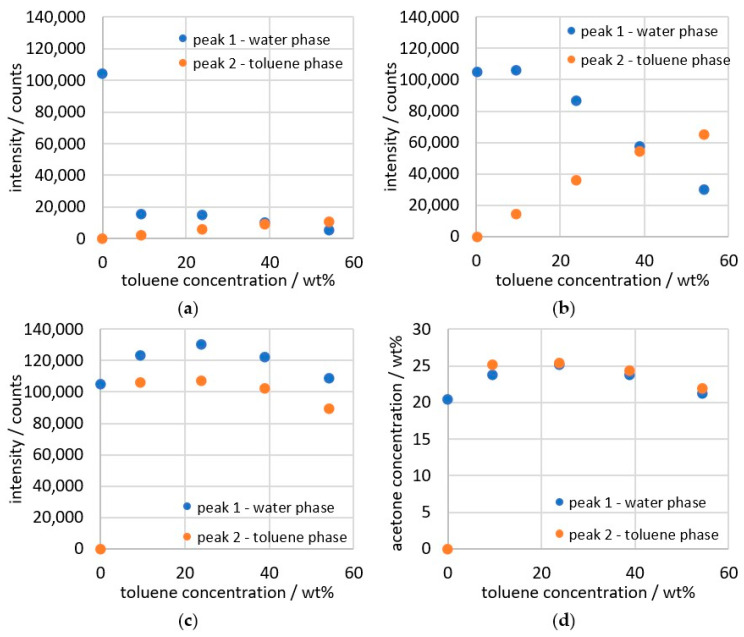
Results of the several calculation steps of 20 wt% acetone in toluene/water. (**a**) data from deconvolution; (**b**) data from scattered light correction; (**c**) data from phase ratio correction; (**d**) data calculated as concentration.

The measurement data obtained from the deconvolution (Figure 8a) of the peaks cannot be used on their own to calculate the concentration, as they are influenced by light scattering from the droplets in the emulsion. Therefore, the established scattered light correction from our previous study [25] must be applied at this point. The scattered light signal that was recorded alongside the Raman measurement is inserted into the quadratic equations (Equations (1) and (2)), which was derived from the correlation between the change in peak height (∆*I*) and light scattering (*S*). The correction was established through the change in peak height, but calibration via the integral would lead to the same factors. Nevertheless, correction through peak height is used here in order to be consistent with previous publications and to have a correction based on independent data sets. The correction factor ∆*I* can then be calculated with the data from the deconvoluted peaks (Equation (3)) to obtain the corrected intensity.

(1) $${∆I}_{S<0.49}=37.23\cdot S^{3}+20.20\cdot S^{2}-1.00\cdot S+1$$

(2) $${∆I}_{S>0.49}=669.29\cdot S^{2}-753.92\cdot S+218.34$$

(3) $$I_{a}=I_{a,\;un}\cdot ∆I$$

The result of the data calculation with the scattered light correction is shown in Figure 8b. An ideal result would provide constant values for both phases that are just above and below the value of the water phase at 0 wt% toluene. However, as can be seen, there is still a change in the measurement data as the toluene content increases. The peaks are significantly influenced by the amount of the phase in which they occur. Since the intensity signal initially only measures absolute proportions, a measurement value of less than 20,000 counts is obtained for the toluene phase at 9.4 wt%, even though the relative proportion in the toluene phase should actually be the same in all data points of the measurement series. In order to determine the concentration in the phases, the volume ratio of both phases to each other in the measuring point must be taken into account. In Equations (4) and (5), the phase ratio is included by the proportion of water and toluene in percent by volume. Since toluene and water are nearly immiscible and no deconvolution is necessary, the concentration of toluene and water can be determined normally via the spectrum or directly via the weighed sample, calculated with the density.

(4) $$I_{a,\;w,\;\mathrm{corr}}\;=I_{a,\;w}\;\cdot \;\left(1+\frac{v_{t}}{v_{w}}\right)$$

(5) $$I_{a,\;t,\;\mathrm{corr}}\;=I_{a,\;t}\;\cdot \;\left(1+\frac{v_{w}}{v_{t}}\right)$$

The data can then be converted into concentrations using Equations (6) and (7) from Figure 3. The result of the conversion is shown in Figure 8.

(6) $$\omega_{a,\;w}\;=-3.4\cdot 10^{-11}\cdot {I_{a,\;w}}^{2}+1.9\cdot 10^{-4}{\cdot I}_{a,\;w}+0.6$$

(7) $$\omega_{a,\;t\;}=-1.5\cdot 10^{-10}\cdot {I_{a,\;t}}^{2}+2.3\cdot 10^{-4}{\cdot I}_{a,\;t}+2.4$$

Figure 6 indicates that the data points for the emulsion should be between 19 and 21 wt% for a measurement series with 20 wt% acetone. The measured data in Figure 8d are approximately 4 wt% above the expected result at up to 25 wt%. The ratio of the water phase to the toluene phase yields a value of 0.95, deviating only 2.96% from the expected value. This shows that the phase ratios can be determined with high accuracy from the measurements of the emulsion. In order to additionally determine the absolute concentration more accurately, the deviation of the data points must be examined more closely.

**Figure 9:**
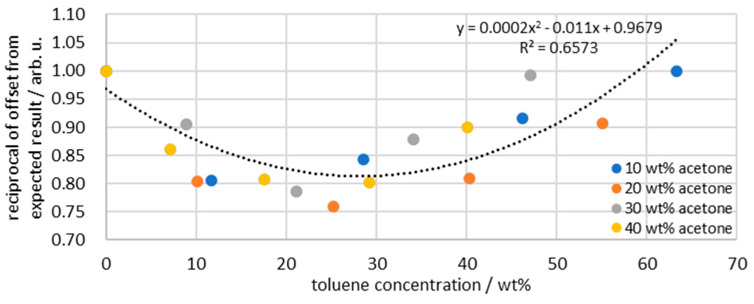
Reciprocal of the deviation of the correction data from the expected data from the separation tests in relation to the toluene concentration; dashed line indicating regression of the data points.

All four data series (10, 20, 30, and 40 wt% acetone) are calculated using Equations (1)–(7), and then the reciprocal of the deviation from the expected values in Figure 6 is determined. The result displays the factors required to correct the measurement data. Plotting all factors together in Figure 9 shows a deviation that occurs systematically in all data series. Due to its systematic occurrence, one cannot assume that it is a measurement error or random error, but rather a physical effect. The assumption is that although the scattered light corrects for light losses, the data changes after application of the deconvolution, and thus the correction fails to produce accurate results. While scattered light correction considers the losses of the entire emulsion, deconvolution leads to a more differentiated representation of the individual peaks. Since both peaks have the same offset, changing the scattered light correction using individual signals does not provide a solution. Influences caused by volume contractions of the individual phases are considered by calibrating and converting the intensities into concentrations. However, it is possible that other molecular interactions occur that lead to signal deviations and are not detected by the scattered light, phase ratio, or concentration calibration. The exact reason for the offset cannot be definitively determined, but due to its systematic occurrence, it is possible to use the regression from Figure 9 to calibrate the measurement system.

**Figure 10:**
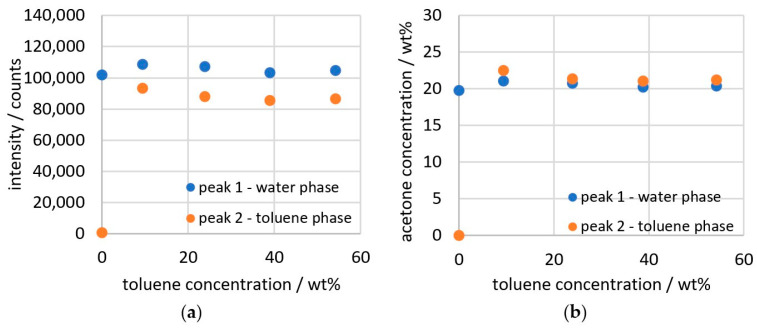
Result of applying the offset correction (**a**) in intensity values and (**b**) in concentration values.

When the regression from Figure 9 is used for correction, the result is shown in Figure 10a and the result converted to concentration is shown in Figure 10b. The average deviation from the expected value for the measurement series with 20 wt% acetone is approximately 1.01 wt%.

**Figure 11:**
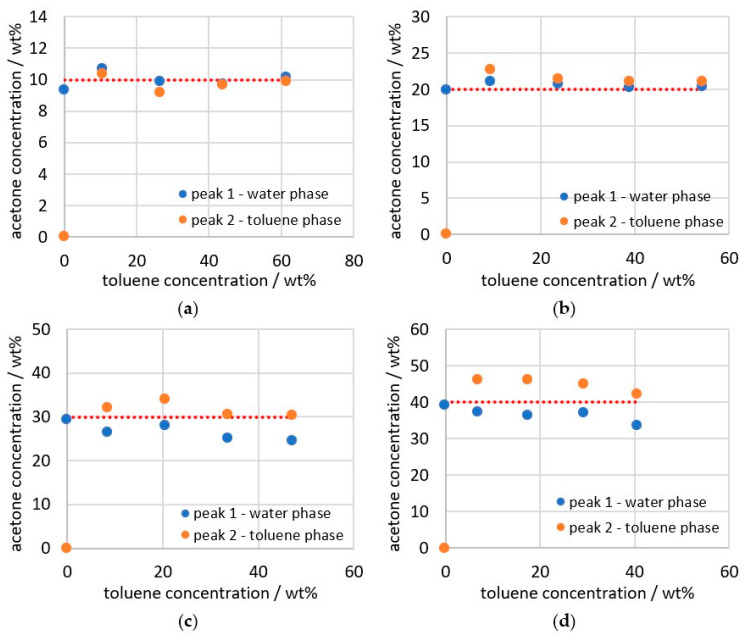
Acetone concentration of the individual phases calculated from the deconvolution for (**a**) 10, (**b**) 20, (**c**) 30 and (**d**) 40 wt% acetone; dashed line indicates the acetone concentration of the target parameter as a reference.

The correction is also done for all other data points and then plotted, as shown in Figure 11. The dashed red line shows the acetone concentration of the total mixture. The highest deviation from the expected concentrations (Figure 6), is 2.64 wt% and can be observed at 30 wt% acetone and 47 wt% toluene. When looking at all the measurement data, it can be seen that, as in Figure 5 and Figure 6, the acetone concentration in the watery phase is higher at 10 wt% acetone (Figure 11a), while at higher acetone concentrations, the toluene-rich phase has a higher acetone content, compared to the water-rich phase.

**Figure 12:**
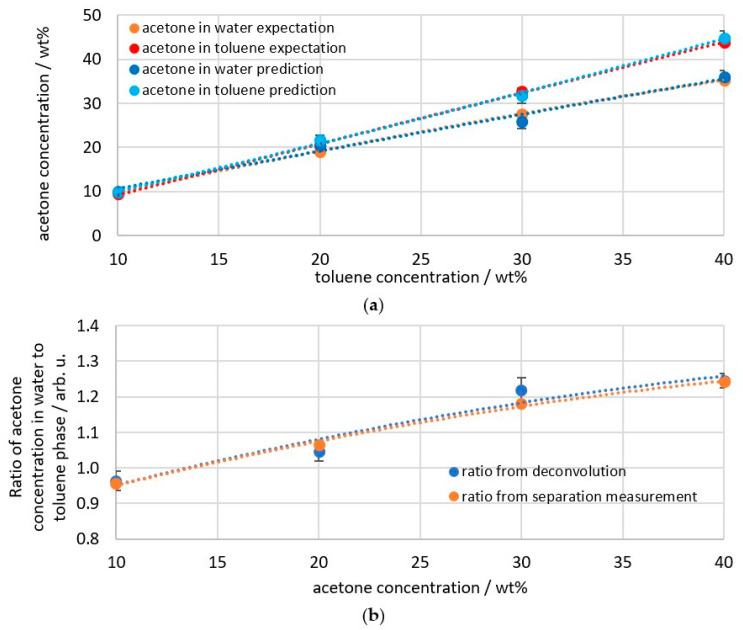
The mean values of the measurement series are shown in comparison with the expected values from the separation experiments regarding (**a**) the absolute concentrations and (**b**) the ratio of the acetone concentration in the water and toluene phases; dashed line indicating regression of the data points.

The mean values of the four measurement series can then be compared with the expected values for the individual phases (Figure 12a). The expected values are the measured values obtained during phase separation. They were therefore measured as a homogeneous mixture after reaching a state of equilibrium. Accordingly, no scattered light correction or deconvolution was applied to the data, only conversion to weight percent as shown in Figure 3. These expected values were also verified using data from the literature [29,36,37]. The largest deviation occurs at 30 wt% acetone and amounts to a *RMSEP* (Root Mean Squared error of Prediction) of 1.74 wt%. The largest relative deviation of 6.8% occurs at 20 wt% acetone. When comparing the phase ratios of the deconvolution and phase separation from Figure 5 and Figure 6, it can be seen that these show only minimal deviations (Figure 12b). Within a series of deconvolution measurements, the values varied by a maximum of ±3.07% at 10 wt% acetone and showed the greatest *RMSEP* from the phase separation measurement at 30 wt% acetone, with a deviation of 0.04 for the ratio. Overall, the *RMSEP* of the absolute measured values from the expected values is 1.3 wt%, and the *RMSEP* of the phase ratio from the expected values is 0.03. The *RMSEP* values calculated using Equation (8) [38] are listed in Table 2 for an overview of the correction accuracy.

(8) $$RMSEP=\sqrt{\frac{1}{N}\cdot \sum_{i=1}^{N}{\left(y_{i}-{\bar{y}}_{1}\right)}^{2}}$$

**Figure 13:**
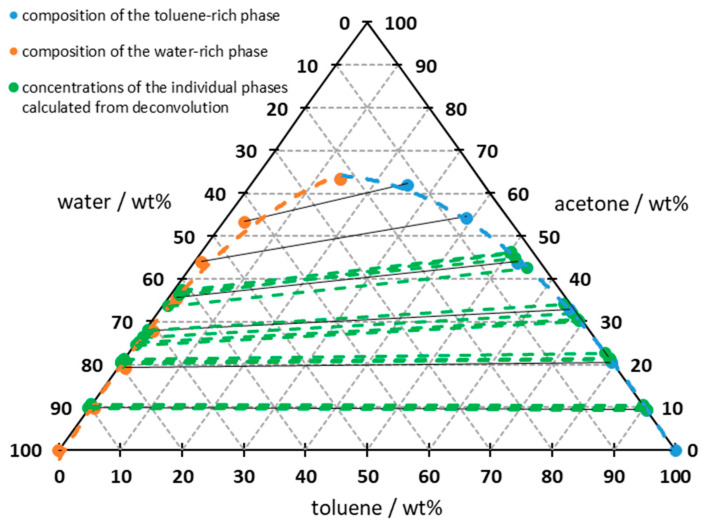
Ternary diagram of water–toluene–acetone with plotted results of the deconvolution; measured at 24 ± 1 °C; dashed line indicating regression of the data points; solid line connecting data points indicates the conodes from phase separation measurement.

In the final step, the individual data points shown in Figure 11 are plotted in the ternary phase diagram (Figure 13) with their conodes in the two-phase region for comparison reasons. The data points for the acetone concentration in each phase are plotted on the corresponding binodal curve. Although the water and toluene proportions of the individual phases cannot be determined from the spectrum, as there is no comparable phase-dependent peak shift as with acetone, the concentrations can still be determined by combining the acetone concentration and the binodal curve. It can be seen that all conodes from the deconvolution run parallel to their nearest conodes from the phase separation or follow the trend of all conodes. This demonstrates that scattered light correction provides a way to overcome interference in a mixed emulsion and helps to determine the acetone concentration in both the dispersed and continuous phases simultaneously.

## 4. Discussion

The aim of the investigations presented here was to use the scattered light correction established in our previous work to distinguish the acetone concentration in the dispersed and continuous phases of a water–toluene–acetone emulsion. The determination of the total acetone concentration in an emulsion has already been successfully applied in the past [25]. The basic signal obtained from a spectrum is sufficient for our purpose. Signal losses can be corrected by quantifying the scattered light. In this way, the scattered light signal offers the advantage that all concentrations and particle sizes lie on the same calibration curve. Therefore, no detailed knowledge of the dispersed phase is necessary, provided that the distribution of the droplets can be assumed to be homogeneous or that stirring ensures that the measuring point provides a representative result for the entire sample.

As shown here, however, scattered light correction can also be used to differentiate between the acetone concentration in the dispersed and continuous phases. This is possible because there is a peak shift depending on which component the acetone is present in. When acetone is dissolved in water, the Raman shift is at 1704 cm^−1^, and when it is dissolved in toluene, it is at 1720 cm^−1^. The dependence of the peak position on the solvent has been reported in several studies. This is due to intermolecular interactions, whereby the Raman shift is influenced by the strength of the bonding forces between the functional groups [39,40,41]. In homogeneous mixtures, there is only one peak, which can shift due to intermolecular forces. In an emulsion, however, each phase produces its own spectrum. As a result, there are two acetone peaks, each subject to different intermolecular binding forces. Because Raman spectroscopy detects both phases simultaneously, the two peaks overlap, and a double peak forms in the observed spectrum. This cannot be differentiated based on peak height alone, since the two acetone peaks influence each other. However, a mathematical deconvolution can be performed that assumes both peaks to be normally distributed and iteratively adjusts them to the measurement curve of the total signal. The two peaks obtained are free of cross-interference from their flanks and can then be corrected for signal losses using scattered light correction. However, the generated data alone does not yet deliver the expected result, because only the absolute proportion of the acetone content in the phases is represented in the spectrum. In order to calculate a concentration, knowledge of the ratio of the dispersed to the continuous phase is necessary. This can either be obtained from the measured weights, or it is necessary to evaluate more wavelengths in the spectrum. For industrial applications, it therefore depends on whether the water and toluene content is known and only the acetone content and its distribution across the individual phases need to be determined, or whether all parameters are unknown or variable, meaning that a peak for toluene and water must also be measured and calibrated. Since toluene has a significantly stronger Raman signal and many easily distinguishable peaks, its overall concentration in the emulsion can be easily determined.

An issue for the correct determination of concentration is that all data display a systematic offset that could not be linked to a specific effect within the scope of our work. However, due to its systematic occurrence, it is possible to calibrate the effect into the data series. It is assumed that these are more complex interaction forces that do not directly influence the scattered light but lead to a change in the Raman effect. This can only be observed in the emulsion, as the signals of the single phase were measured and calibrated for the mixture. Thus, effects such as volume contraction between acetone–water and acetone–toluene were also taken into account. Theoretically, however, the offset correction step can be skipped entirely if knowledge of the ratio of acetone concentration in the water phase to acetone concentration in the toluene phase is sufficient. This is because even if the determination of the acetone concentration provides results that are too high, the ratio of the concentrations to each other corresponds to the expected values. With knowledge of the ternary diagram, it would then be possible to draw conclusions about the absolute concentrations.

After these calculations, concentration values were obtained which, when plotted in a ternary phase diagram, gave representative and reproducible conodes. The ratio of the acetone content in the toluene to the water phase corresponded to measurements from phase separation, which are considered plausible based on comparisons with the literature. It can therefore be concluded that the method was successfully applied to demonstrate a new method for monitoring emulsions with regard to their diffusion processes. This may make it possible in the future to simplify process control by eliminating the need for phase separation cells, for example. The method also offers easier access for basic investigations of new chemical systems, for example, as light losses due to dispersed phases are no longer a problem.

## 5. Conclusions

It has been shown that the scattered light correction method can not only determine the total concentrations of an emulsion but is also capable of distinguishing between components that are soluble in both phases. Determining the total concentration of a component in an emulsion requires only information from individual wavelength ranges and can easily be performed using photometric measurement methods. However, for the determination of the concentration in individual phases a spectral measurement of the double peak range is required in order to perform the deconvolution. Knowledge of the phase ratio is also necessary, which requires either knowledge of the added components or photometric determination of other components of the emulsion. Determining the concentration in the individual phases therefore requires greater analytical effort but also provides a much more detailed insight into the emulsion. The result ultimately provides valuable information about the distribution of acetone in the dispersed and continuous phases and can therefore be used to track diffusion between the phases and/or to determine whether stable states have been reached. The method was designed as a general approach. The basic concept of scattered light correction should therefore be applicable to all mixtures that exhibit an evaluable peak in the Raman spectrum. The same limitations apply here as for Raman measurements of homogeneous mixtures. The peak of the substance to be determined must not be overlapped and must be correlatable with the change in concentration. If this is ensured, the substance can also be measured in an emulsion using scattered light correction. However, the method presented here for additionally determining the concentration in the individual phases is more specific and has the additional requirement that a peak shift occurs, which allows for a differentiation. In principle, the peak position always depends on the overall mixture, and molecular interactions can cause a shift in position. However, it must always be verified whether the shift is large enough to allow for peak deconvolution. The conclusion is therefore that a basic scattered light correction is always applicable whenever regular Raman spectroscopy would also be possible in the homogeneous phases. For the extended correction, a double peak must be present that can be deconvoluted, which must be verified beforehand.

## Figures and Tables

**Table 1:** Prepared samples to measure the Raman spectrum with scattered light correction.

Water/wt%	Toluene/wt%	Acetone/wt%	Polysorbate20/wt%
87.86	0.00	10.08	2.05
77.44	10.56	10.00	2.00
61.35	26.46	10.04	2.14
43.78	43.81	10.24	2.17
26.31	61.48	10.04	2.17
78.22	0.00	19.69	2.09
68.66	9.42	19.92	2.01
54.08	23.85	20.11	1.97
38.70	38.91	19.89	2.49
23.24	54.29	20.14	2.33
67.84	0.00	29.97	2.19
59.27	8.42	29.92	2.40
47.68	20.43	29.81	2.08
34.18	33.54	30.25	2.03
20.79	47.10	30.07	2.04
57.84	0.00	39.96	2.20
51.17	6.96	39.78	2.09
40.67	17.32	39.86	2.16
28.81	29.26	39.82	2.12
17.35	40.71	39.76	2.18

**Table 2:** RMSEP values of the results of the correction.

Acetone Conc./wt%	RMSEP of Conc./wt%	RMSEP of Ratio/-
10	0.44	0.03
20	1.16	0.02
30	1.74	0.04
40	1.49	0.02

## Data Availability

The data presented in this study are available on request from the corresponding author.
